# Intelligent Testing Method for Multi-Point Vibration Acquisition of Pile Foundation Based on Machine Learning

**DOI:** 10.3390/s25092893

**Published:** 2025-05-03

**Authors:** Ke Wang, Weikai Zhao, Juntao Wu, Shuang Ma

**Affiliations:** 1 College of Civil Engineering and Architecture, Zhejiang University, Hangzhou 310058, China; 3110100354@zju.edu.cn (K.W.); zhaoweikai24@163.com (W.Z.); 2 School of Spatial Planning and Design, Hangzhou City University, Hangzhou 310015, China

**Keywords:** pile foundation, low strain, high-cap, multi-point vibration acquisition, machine learning

## Abstract

To address the limitations of the conventional low-strain reflected wave method for pile foundation testing, this study proposes an intelligent multi-point vibration acquisition testing model based on machine learning to evaluate the integrity of in-service, high-cap pile foundations. The model’s performance was assessed using statistical error metrics, including the correlation coefficient R^2^, mean absolute error (MAE), and variance accounted for (VAF), with comparative evaluations conducted across different model frameworks. Results show that both the convolutional neural network (CNN) and the long short-term memory neural network (LSTM) consistently achieved high accuracy in identifying the location of the first reflection point in the pile shaft, with R^2^ values greater than 0.98, MAE below 0.41 (m), and VAF greater than 98%. These findings demonstrate the model’s strong predictive capability, test stability, and practical utility in supporting operator decision-making. Among the evaluated models, CNN is recommended for analyzing the integrity of in-service pile foundation based on the multi-point vibration pickup signals and multi-sensor fusion signal preprocessed by the time series stacking method.

## 1. Introduction

Pile foundations, as the main load-bearing components supporting superstructures, directly affect the safety of major infrastructure such as ports, terminals, high-speed railways, and high-rise buildings [1]. For example, a magnitude 7.4 earthquake occurred in Maduo County, Golog Tibetan Autonomous Prefecture, Qinghai Province on 22 May 2021, causing varying degrees of damage to the pile foundation of the Yematan Bridge, resulting in continuous collapse of multiple spans of the bridge. Therefore, it is necessary to evaluate the integrity and health status of service pile foundations during long-term operation and post-disaster reinforcement and repair stages to ensure pile foundation quality and reduce safety hazards [2].

Various nondestructive testing techniques have been proposed to evaluate the integrity of foundation piles, including the low-strain wave method [3], cross-hole sonic logging [4], high-strain dynamic testing [5], core-drilling, and parallel seismic methods [6]. Among these, the low-strain reflection wave method has been widely adopted in engineering practice due to its operational simplicity and cost-effectiveness [7]. In recent years, extensive research has been devoted to this area, yielding significant advancements. For example, Wang et al. [8] investigated the vertical dynamic response of an inhomogeneous viscoelastic pile embedded in layered soil and obtained the analytical solutions of pile responses in the frequency domain by using the Laplace transform. Cui et al. [9] derived an analytical solution for the dynamic impedance at the top of pipe piles embedded in a viscoelastic soil layer with radial inhomogeneity by extending Novak’s plane strain model. Fu et al. [10] proposed a defective pile–porous fictitious soil pile-saturated layered soil coupled vibration system to investigate the response of the fully saturated soil around and beneath the vibrating pile with various defects. Zheng et al. [11] studied the effect of radial soil deformations on the dynamic response of floating piles and considering both vertical and radial soil displacements generated by axial pile vibration.

However, when the pile to be tested is located beneath an existing structure, the traditional low-strain reflection wave method becomes significantly compromised. The resulting waveforms are often distorted, making it difficult to interpret the data and accurately assess the integrity of the pile body [12]. Furthermore, the existing test theory usually fails to provide accurate solutions under such conditions, often yielding degenerate or approximate results that deviate markedly from the real-world engineering situation [13]. To address these limitations, Wu et al. [14] introduced the Multi-point Traveling Wave Decomposition Method (MTWDM), which effectively mitigates the dynamic interference from the superstructure and enables integrity assessment of in-service high-cap pile foundations. Despite its advantages, the MTWDM is highly dependent on the quality of traveling wave decomposition process. Moreover, the method is limited to qualitative evaluations and cannot provide fully quantitative assessments, leaving the accuracy of test results reliant on the subjective judgment and experience of the operator.

Data-driven artificial intelligence algorithms have gained increasing traction in geotechnical engineering applications [15]. Numerous researchers employed machine learning (ML) algorithms to automatically predict landslide susceptibility [16], ground displacement [17], and Lithology classification and identification [18]. However, the application of ML to pile integrity evaluation and defect detection remains relatively underexplored. The key advantage of ML algorithms lies in their ability to extract complex, high-order patterns from data [19]. For example, convolutional neural networks (CNNs) can automatically capture the implicit spatial correlation and scale invariance features in the data by constructing multi-scale feature extraction modules [20]. Given that the MTWDM has achieved signal reconstruction from a theoretical level and has been successfully applied to the integrity detection of high-cap pile foundations in service, its multi-sensor layout should contain almost all the mechanical information of the piles to be tested below the measuring point. This provides a strong foundation for ML models to learn from fused multi-sensor data and predict the mechanical behavior and service condition of pile foundations beneath existing structures.

Based on the above background, this study aims to address the limitations of the traditional low-strain reflection wave method, which relies on single-channel vibration signals. In such methods, the vibration of the upper structure is superimposed on the vibration of the pile body, making it difficult to judge the quality of the pile body and restricts assessment to a qualitative level. To overcome these challenges, this paper adopts the optimized multi-sensor layout scheme of the MTWDM and innovatively proposes a damage detection model for in-service pile foundations beneath existing structures. The proposed model leverages multi-sensor data fusion and machine learning algorithms to enable more accurate and quantitative assessment of pile foundation integrity.

Unlike most intelligent detection models based on the traditional low-strain reflection wave method that is typically limited to classifying the severity of pile body defects [21], the multi-sensor array acquisition method employed in this study enables information complementarity and fusion across channels. This allows the model to extract more comprehensive quality indicators from the pile body, enabling not only the prediction of potential defects but also the estimation of the pile bottom position. In doing so, the proposed method supports the quantitative analysis of pile integrity, advancing beyond the qualitative capabilities of earlier models. In contrast to existing models that rely on small and often noisy real-world inspection datasets [22], this study simulates inspection data under diverse working conditions through analytically derived solutions. This strategy facilitates the development of a large-scale, high-quality database that ensures more robust model training and validation.

In order to effectively integrate information from multiple sensors, this paper employs three different preprocessing techniques: time series splicing, time series stacking, and time-frequency stacking. The impact of each method on model performance is systematically evaluated to identify the optimal combination of data preprocessing strategy and model architecture for quantitatively assessing pile foundation integrity. The findings from this research offer valuable and novel insights for the rapid assessment of the health status of pile foundations in existing structures, particularly during long-term service or following seismic or other disaster-related events.

## 2. Multi-Point Traveling Wave Decomposition Method (MTWDM)

For the integrity detection of high-cap service pile foundations under existing structures, due to the interference of upper structure vibrations on the test results, it is difficult to obtain effective information from the pile top velocity response curve obtained by the traditional low-strain reflected wave method to judge the quality of the pile shaft. To overcome the limitations of existing pile foundation quality testing methods, Wu et al. [14] proposed the MTWDM based on traveling wave decomposition theory.

**Figure 1:**
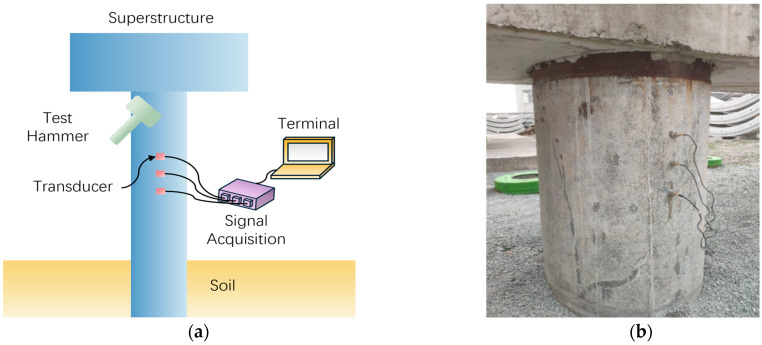
Schematic diagram of MTWDM: (**a**) test system diagram; (**b**) instructions for use in actual projects.

This method first measures the wave velocity of the pile foundation to be tested through the distance between two sensors and the time difference of the first wave. As shown in Figure 1, three sensors are arranged at equal intervals on the exposed section of the pile shaft (spacing ≤ 0.5m and an integer multiple of the product of wave velocity and sampling interval), and the test excitation consistent with the direction of the sensor reception is applied directly above the measurement point, and multi-channel signals are collected synchronously. Then, based on traveling wave decomposition theory, the upward and downward wave components are extracted, and the generalized frequency response function is defined as the ratio of the upward and downward wave functions in the frequency domain. Finally, virtual test excitation is introduced to obtain the reconstructed pile shaft response curve below the measurement point.

This method has the following advantages for the integrity detection of service pile foundations under existing structures:

(1) The reconstructed results can effectively eliminate the interference of upper structure vibrations above the measurement point while retaining all the mechanical information of the pile foundation below the measurement point;
(2) The reconstructed signal is close to the results of the traditional low-strain reflected wave method, reducing the learning cost of the detection personnel;
(3) The equipment is lightweight, only requiring a hammer, sensors, signal acquisition device, and a receiving terminal for detection, suitable for general surveys of high-cap service pile foundations.

However, like the traditional low-strain reflection wave method, the MTWDM still relies heavily on the professional experience of the detection personnel, only achieving qualitative judgment on the integrity of the pile shaft, and it is difficult to form quantitative conclusions [23]. In addition, due to the mechanical error of the equipment, the results based on theoretical reconstruction inevitably have systematic bias. Given that the sensor layout scheme of MTWDP has theoretically achieved signal reconstruction and is used for the integrity judgment of high-cap piles, its sensor layout can theoretically effectively capture the vibration characteristic information of the pile shaft. Therefore, this paper proposes an intelligent detection model that can avoid theoretical analysis and directly analyze and judge the distance from the first reflection point to the middle measurement point in the signal of multiple sensors based on machine learning algorithms and multi-channel acquisition technology of multi-point vibration acquisition. Compared with the traditional single-point reception test mode of low-strain reflected wave detection of pile foundations, the MTWDM based on distributed sensor arrays can achieve dense spatial domain sampling of pile shaft vibration signals, with complementary information between different sensors, allowing ML algorithms to extract richer pile shaft quality features, such as the wave velocity information of the pile foundation stored in different channels at the same sampling point.

## 3. MTWDM Results Database

### 3.1. Database Generation

Given the difficulties in data acquisition and high cost of pile integrity detection in actual projects [24], and the fact that the measured signals are easily disturbed by instrument drift and environmental noise, existing technologies have difficulty identifying all defects in the pile body [25], resulting in reduced data quality and label reliability. Hence, this paper adopts analytical solutions with less data noise and better data quality to establish a training and verification database to prevent the interference signals in the real data from causing the model to learn incorrect kinematic characteristics of pile vibration. Since Wu et al. [14] have proposed an analytical model for the MTWDM and verified that the proposed model is consistent with the actual working conditions, this paper adopts the analytical model proposed by Wu et al. to simulate the multi-channel pile foundation vibration response signals collected by three velocity sensors arranged at equal intervals on the section of the pile body exposed to the ground. In addition, random white noise is superimposed on each generated data to simulate the mechanical error caused by the instrument in the actual detection, thereby improving the generalization performance of the model.

To construct a high-quality training dataset and improve the generalization performance of intelligent algorithms, this paper adopts a systematic parameter control strategy [26] to generate uniformly distributed theoretical samples, with parameters that can randomly vary within a limited range including but not limited to various parameters of soil and pile shaft, defect segment position and length, pulse width, and other key variables; the variation ranges for each parameter are shown in Table 1.

**Figure 2:**
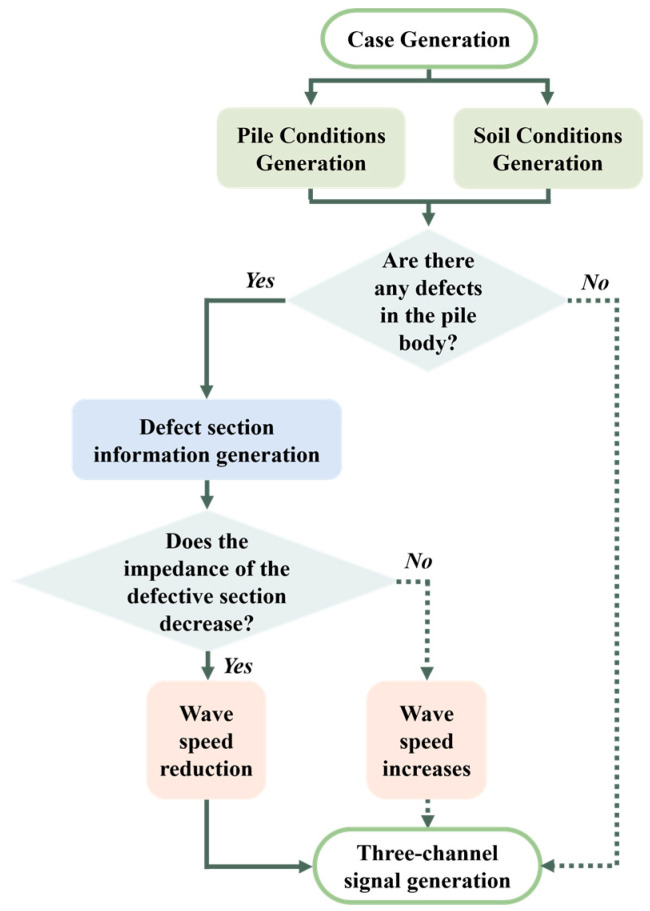
Flow chart of case generation.

In addition, the dataset comprehensively covers the working conditions of intact piles, piles with reduced impedance defects (such as necking, concrete segregation, mud inclusions, etc.), and piles with increased impedance defects (such as diameter expansion). In the analytical solution model, the simulation of different pile foundation integrity conditions is mainly achieved by changing the longitudinal wave velocity of the pile body [27]. Specifically, the longitudinal wave propagation velocity of the intact pile section is between 3000 and 4000 m/s [28]; the longitudinal wave propagation velocity of the impedance-reduced defect pile section is set between 1000 and 2000 m/s [29]; and the longitudinal wave propagation velocity of the impedance-increased defect pile section is between 5000 and 6000 m/s [30]. The generation process flowchart under different conditions is shown in Figure 2.

**Figure 3:**
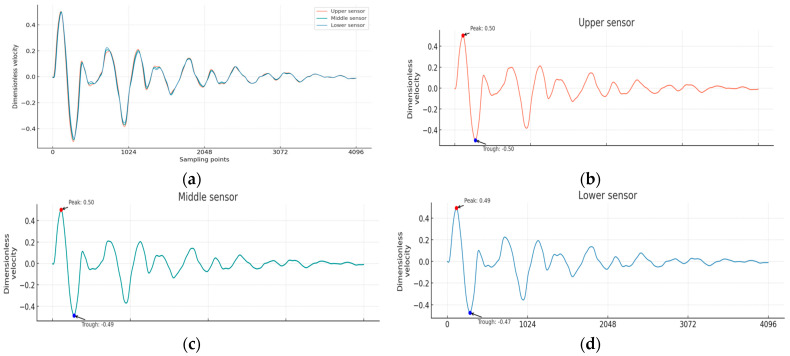
The MTWDM results simulated by analytical solution under typical cases: (**a**) the comparison of signals collected by three sensors; (**b**) signal collected by the upper sensor; (**c**) signal collected by the middle sensor; (**d**) signal collected by the lower sensor.

Figure 3 shows the multi-channel vibration response time history curves simulated by analytical solutions under typical conditions. In the analytical solution, generalized incident waves are introduced to normalize the generated results to the [−1, 1] standard interval, effectively improving the model calculation and convergence speed [31]. Finally, the dataset is divided into two parts, with 80% of the data used to train the model and the validation part using 20% of the data [32].

### 3.2. Data Preprocessing

To achieve efficient analysis and feature extraction of multi-channel sensor signals by the calculation model, this paper integrates multi-sensor information from the perspective of data dimensions using three data preprocessing methods to provide high-quality input for the model, with specific methods as follows.

**Figure 4:**
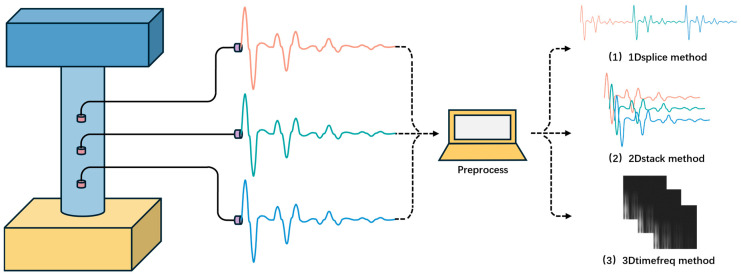
Data preprocessing methods.

(1) Time series splicing method (1Dsplice): As shown in Figure 4 (1), the time sequence data collected by a single sensor are stored in a one-dimensional floating-point matrix with a size of [4096], and then three groups of matrices are sequentially spliced into a one-dimensional input tensor with a size of [12288]. This method has a simple integration process without requiring a large amount of data processing, but it is difficult to fully extract the complex information inherent in the system.
(2) Time series stacking method (2Dstack): As shown in Figure 4 (2), the time sequence data collected by a single sensor are stored in a two-dimensional floating-point matrix with a size of [1, 4096], and then three groups of single-channel matrices are stacked into a two-dimensional input tensor with a size of [3, 4096]. This method fully retains the time-domain continuity of the pile shaft vibration signal, inputting all pile shaft information collected by the sensors into the model. However, the high-frequency multi-channel signal acquisition leads to a significant increase in the model calculation complexity, which can be effectively alleviated by the spatial down-sampling operation of the pooling layer.
(3) Time-frequency stacking method (3Dtimefreq): As shown in Figure 4 (3), the time-domain pile shaft vibration signal of a single sensor is decomposed into multi-scale through continuous wavelet transform (CWT), generating a two-dimensional feature map containing scale-time spectrum information. To balance information retention and calculation efficiency, this paper uses 64 × 64 pixel resolution and only extracts grayscale features. Finally, three groups of images are stacked into a three-dimensional input tensor with a size of [3, 64, 64] along the channel direction. This method enhances the feature dimension from one-dimensional time-domain signals to three-dimensional frequency-domain tensors, facilitating the model to capture multi-scale velocity response features in the signal.

## 4. Calculation Model

With the integration and innovative development of artificial intelligence algorithms and the field of geotechnical engineering, intelligent calculation models based on ML algorithms continuously demonstrate advantages in key aspects such as geotechnical parameter inversion and engineering stability evaluation. Deep neural network models can capture complex mathematical relationships and high-order statistical patterns beyond human cognition from information, effectively identifying non-intuitive data patterns that are difficult to recognize by traditional methods. Based on this, this paper proposes CNN models and LSTM models to achieve intelligent detection of the integrity of high-cap service pile foundations under existing structures.

### 4.1. Convolutional Neural Network (CNN) Model

Convolutional neural networks are a class of feedforward neural networks that include convolution operations. The traditional CNN structure consists of an input layer, an output layer, and a sequence of hidden layers containing convolutional layers, pooling layers, and fully connected layers [33]. In the convolutional layer, the convolution kernel performs discrete convolution calculations on the input data according to formula (1) to extract feature information [34]. The pooling layer achieves data down-sampling by performing operations such as taking the maximum value or average value of the features obtained by convolution to retain key information [35]. The fully connected layer integrates all features through linear transformation to obtain the output.

(1) $$y_{i,j}=\sigma (W_{k}⊗x_{i,j}+b_{k})$$

(2) $$W_{k}⊗x_{i,j}=\sum_{m=0}^{a-1}\sum_{n=0}^{b-1}w_{m,n}\times x_{i+m,j+n}$$

where *x_i,j_* is the input data of the convolution layer; *y_i,j_* is the output data of the convolution layer; ⊗ is the discrete convolution operation; *W_k_* and *b_k_* are the weight coefficients and bias parameters of the *k*th convolution kernel, respectively; *σ* is the activation function of the neuron; and *a* and *b* are the size parameters of the convolution kernel.

**Figure 5:**
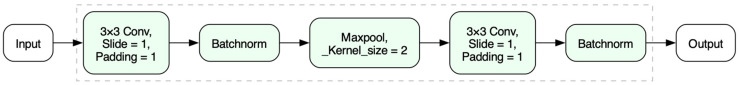
CNN convolutional block structure.

In order to improve the feature extraction capability of CNN for the proposed multi-sensor fusion information, this paper designs a multi-level feature fusion convolution module as shown in Figure 5. In the first layer, the 3 × 3 convolution kernel is used for feature channel expansion, doubling the input channel dimension; the second layer of the same size convolution kernel performs secondary feature extraction while maintaining the channel dimension unchanged, enhancing the robustness of feature representation; the Batchnorm layer implements feature distribution standardization to accelerate model convergence speed; the ReLU activation function introduces a nonlinear transformation mechanism, effectively improving the model’s expressive ability; and the Maxpooling layer performs feature dimensionality reduction operations, retaining the maximum response features while compressing the parameter quantity, thereby reducing calculation complexity.

**Figure 6:**
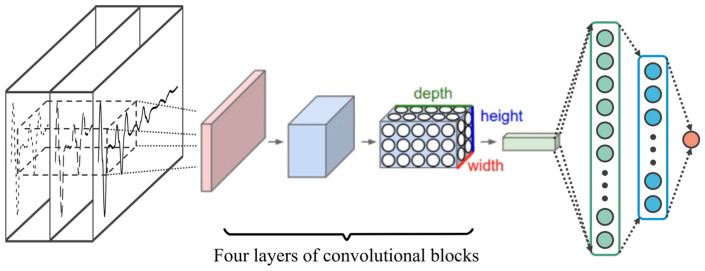
CNN model architecture.

Based on the improved convolution feature extraction block, using the time sequence stacking method to preprocess the input data as an example, the overall architecture of the CNN model proposed in this paper for automatic pile foundation damage detection is shown in Figure 6.

### 4.2. Long Short-Term Memory Neural Network (LSTM) Model

**Figure 7:**
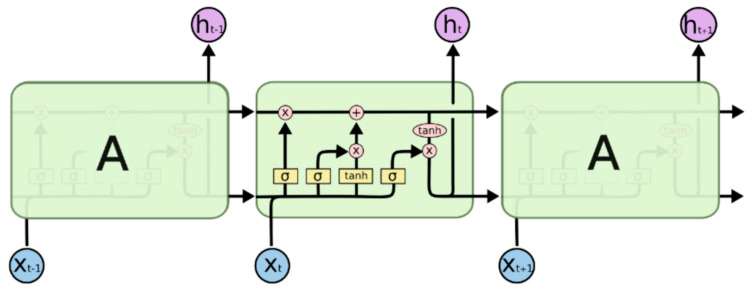
Schematic diagram of LSTM module.

Long short-term memory neural networks are a variant of recurrent neural networks (RNNs) [36]. LSTM neural networks achieve dynamic modeling of long-term and short-term dependencies in time series data by constructing a gating mechanism that includes a forget gate, input gate, and output gate. At the same time, the linear transmission of cell states and the nonlinear transformation of gating units effectively solve the gradient disappearance or gradient explosion problem that occurs in RNNs during back propagation [37]. The structure of the LSTM unit is shown in Figure 7.

**Figure 8:**
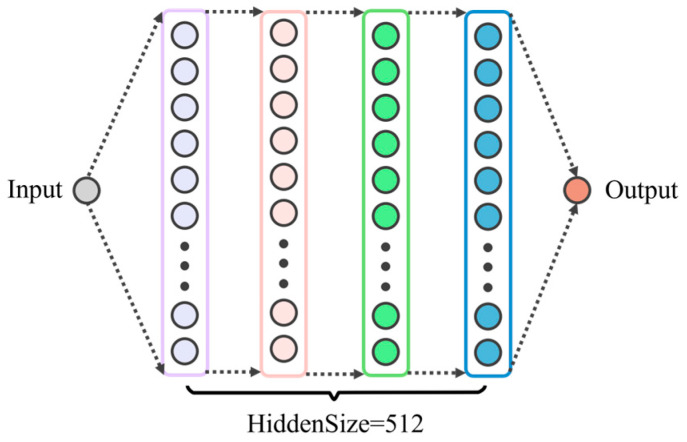
LSTM model architecture.

Using a systematic grid search method to find the optimal hyperparameter combination of the LSTM model [38], the specific parameter space settings are as follows: the number of neurons in the hidden layer is geometrically expanded from 16 to 1024 in powers of 2; the number of hidden layers is sampled at equal intervals with a step size of 2 in the range of [2,8]; and the regularization parameter dropout is searched exhaustively in the range of 0.3–0.7 with a step size of 0.1. In the multi-dimensional parameter space, exhaustive cross-validation is implemented to finally determine the optimal hyperparameter combination. After optimization, the architecture of the LSTM model proposed in this paper is shown in Figure 8.

### 4.3. Loss Function, Optimization, Regularization, and Early Stopping Strategy

The mean absolute error (MAE) is used to measure the difference between the model judgment value and the true value, and its form is as follows:

(3) $$MAE=\frac{1}{n}\sum_{i=1}^{n}\left|y_{i}-{\hat{y}}_{i}\right|$$

where *n* is the number of samples, *y_i_* is the true value of the *i*th sample and is the judgment value of the *i*th sample.

Compared with the widely used mean squared error (MSE), MAE avoids the quadratic amplification effect of the difference in abnormal sample points through a linear penalty mechanism, thereby showing stronger robustness to abnormal values in statistical characteristics [39]. From the perspective of differential characteristics, the gradient of MSE is linearly related to the error magnitude, which causes abnormal sample points to generate excessively large gradient updates, leading to the model parameters deviating from the optimization direction dominated by normal samples [40]. The gradient magnitude of MAE is constant at 1, and this equal-weight update mechanism effectively avoids the gradient dominance problem of abnormal samples. Therefore, both the CNN and LSTM models in this paper use MAE, which has been described in detail in the article as the loss function.

To address the non-convex optimization problem of deep neural networks, this paper uses the Adam optimizer for parameter updates, with an initial learning rate set to 0.0001 [41]. This method achieves dynamic adjustment of the learning rate through the second-order moment estimation mechanism, allowing it to timely adjust the step size according to the gradient changes during the training process to improve optimization efficiency.

To address the problem of parameter redundancy and overfitting in deep neural networks, this paper uses dropout (*p* = 0.7) in the fully connected layer to randomly shield 70% of neuron connections in each training batch [42]. This random topology structure change forces the network to establish redundant feature representations, suppressing the co-adaptation effect between neurons and improving the model’s generalization ability.

L2 regularization (weight decay) is a commonly used parameter regularization method that penalizes the size of the weight parameters in the model to prevent overfitting by adding the sum of the squares of the weight parameters to the loss function [43], and its form is as follows:

(4) $$L=L_{0}+\lambda \sum_{i}^{n}w_{i}^{2}$$

where *L* is the loss function after adding the regularization term, *L*_0_ is the original loss function of the model on the training set, *λ* is the regularization strength, *w_i_* is the *i*th weight parameter in the model, and *n* is the total number of weight parameters.

Since there are many time series signal data points processed in this paper (a total of 3 × 4096 data points), L2 regularization (weight_decay = 0.0001) is used to smooth the model weights so that similar data points have similar weights, thereby effectively avoiding overfitting and improving the generalization of the model [44]. At the same time, To avoid overfitting in the later stages of training, an early stopping strategy [45] is implemented: a validation loss observation window of 20 cycles is set, and if the validation loss does not decrease for 20 consecutive cycles, the training termination mechanism is triggered.

## 5. Model Performance Testing

This paper seeks the optimal intelligent detection model through horizontal comparison between two classic model frameworks and vertical comparison between different data preprocessing methods under the same model framework. The naming of the schemes in the experiment is as follows: 1Dsplice-CNN/1Dsplice-LSTM represents the model framework based on the time sequence splicing method; 2Dstack-CNN/2Dstack-LSTM corresponds to the model architecture using the time sequence stacking method; and 3Dtimefreq-CNN represents the CNN model using the time sequence stacking method.

**Figure 9:**
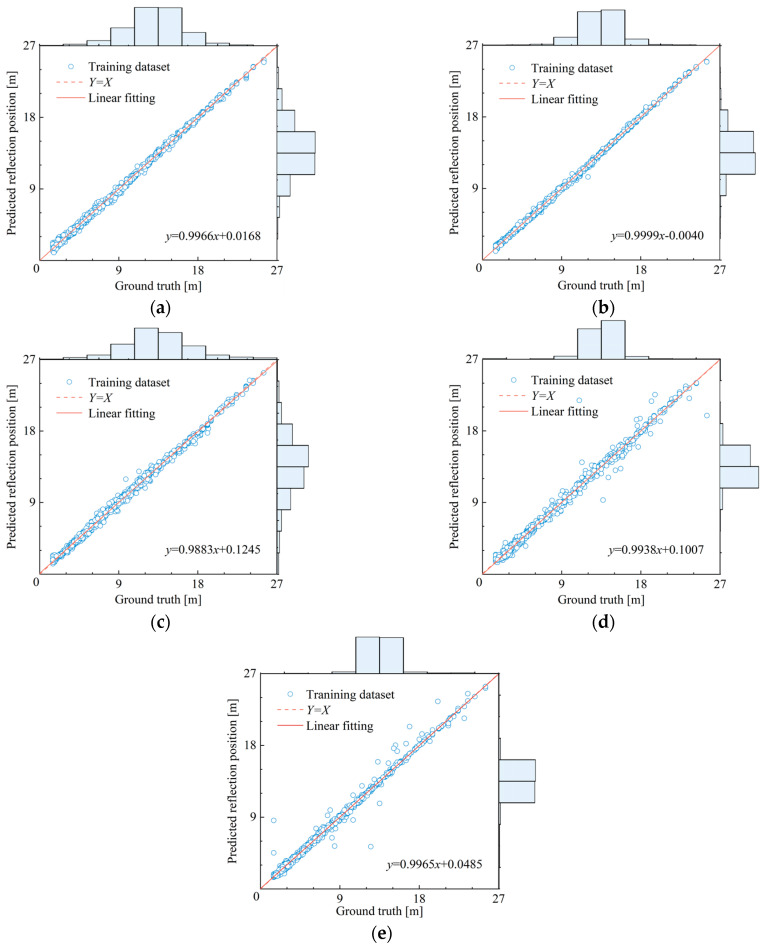
Comparison between the judgment value and the true value of training dataset of models: (**a**) 1Dsplice-CNN; (**b**) 2Dstack-CNN; (**c**) 3Dtimefreq-CNN; (**d**) 1Dsplice-LSTM; (**e**) 2Dstack-LSTM.

As shown in Figure 9, based on the first 1000 sample data of the training set, the matching degree between the judgment value and the true value of the first reflection position output by the detection model were compared and analyzed.

The results show that all model frameworks can effectively capture the main features of the data, but there are significant differences in their fine-grained feature representation capabilities. The CNN architecture exhibits stable feature extraction capabilities under three preprocessing modes, showing excellent fitting performance on the training set; in contrast, although LSTM has advantages in capturing time sequence correlation features, its cross-channel feature fusion capability is weak, leading to underfitting even in the training set. This comparative analysis fully demonstrates the advantages of the CNN architecture in multi-point vibration acquisition intelligent detection. At the same time, both model architectures have achieved a certain degree of improvement in feature representation capabilities under the time sequence stacking method, which also indirectly proves the advantages of multi-point vibration acquisition testing methods in the field of intelligent pile foundation detection.

**Figure 10:**
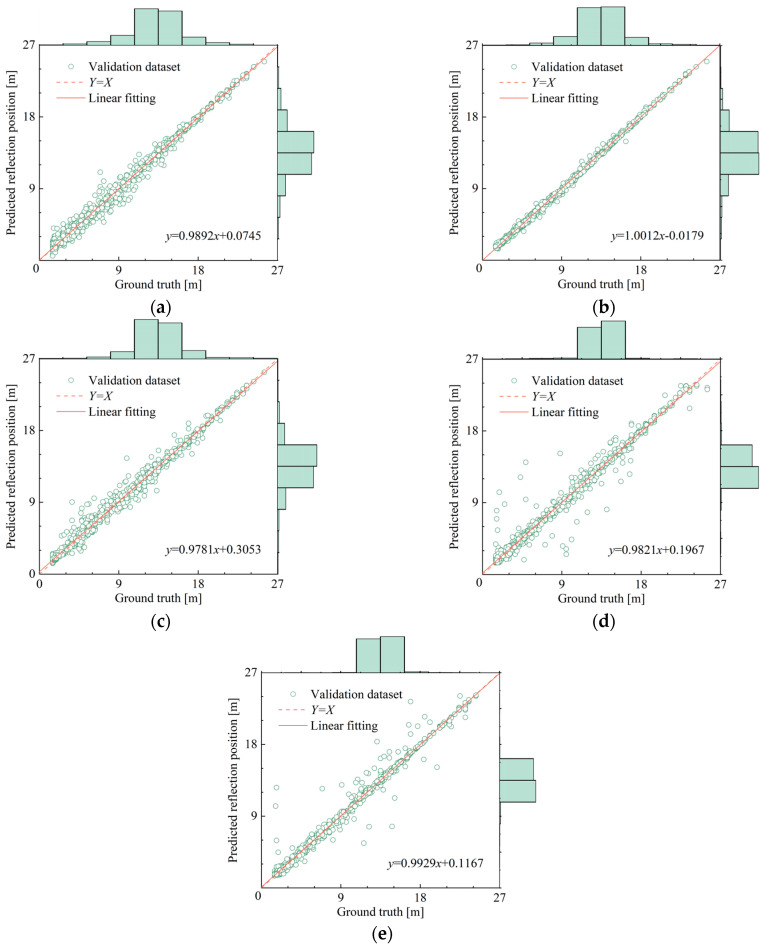
Comparison between the judgment value and the true value of validation dataset of models: (**a**) 1Dsplice-CNN; (**b**) 2Dstack-CNN; (**c**) 3Dtimefreq-CNN; (**d**) 1Dsplice-LSTM; (**e**) 2Dstack-LSTM.

As shown in Figure 10, based on the first 1000 sample data of the validation set, the matching degree between the judgment value and the true value of the first reflection position output by the detection model is compared and analyzed.

From Figure 10, it can be seen that although most model architectures exhibit good fitting performance on the training set, their generalization ability for unseen data shows significant differences. It is worth noting that the 2DStack-CNN model still maintains excellent judgment ability in the validation set, which also verifies the advantages of the CNN architecture in feature extraction. At the same time, the time sequence stacking method effectively enhances the multi-scale feature capture ability of CNN, making the model strengthen global pattern perception while retaining local feature correlation, thereby showing stronger applicability in multi-point vibration intelligent detection tasks. Based on the comparison results, it is recommended to use the 2DStack-CNN as the preferred scheme for multi-point vibration intelligent detection.

To quantify the quality of the calculation model, in addition to the MAE introduced, correlation coefficient R^2^ and variance ratio VAF are also used [46]:

(5) $$VAF=\left[1-\frac{\mathrm{var}(y_{0}-y_{p})}{\mathrm{var}(y_{0})}\right]\times 100$$

where *y*_0_ is the ground true, *y_p_* is the model prediction value, and var is the variance. The results of the three performance indicators of different calculation models are shown in Table 2.

The above conclusions fully verify the feasibility of the new type of damage detection model for serving pile foundations under existing structures based on multi-sensor data fusion and the ML algorithm proposed in this paper, and demonstrate the advantages of the data preprocessing method used. However, since the intelligent detection model proposed in this paper realizes the quantitative judgment of the pile body quality by learning to judge the vertical distance from the intermediate sensor to the position of the first reflected wave in the pile body, when there are multiple defects in the pile body, the model can only judge the position of the defect closest to the vertical distance from the intermediate sensor, but cannot judge the position of other defects. Secondly, this paper simulates defective piles by changing the longitudinal wave propagation velocity of a certain length of pile segment at a certain depth. However, in actual engineering, only a small part of the pile body may have defects at a certain depth. Therefore, for this working condition, the model may have misjudgment problems. Although the model proposed in this paper has the above limitations, the data preprocessing method used, the method of synchronously collecting pile vibration signals with optimized layout of multiple sensors, and the method of generating data under different working conditions by the analytical solution of the model to establish a database can provide a new method for the quantitative and intelligent detection of pile foundation integrity, and expand the application of machine learning algorithms in the field of pile foundation detection, so as to break through the bottleneck of the traditional low-strain reflection wave method and enable it to be applied to the integrity detection of in-service pile foundations under existing structures that are difficult to quantitatively analyze.

## 6. Conclusions

- Based on the CNN model, LSTM model, and three data preprocessing methods, an intelligent detection model for damage to in-service pile foundations under existing structures based on multi-sensor data fusion and an ML algorithm is innovatively proposed, which can quantify the distance between the first reflection point in the signal and the intermediate sensor. The results show that the combination of each model framework and data preprocessing method can effectively complete the task of quantifying pile foundation damage.
- The analytical solution method is used to simulate the multi-channel pile foundation vibration response signal collected by three velocity sensors arranged at equal intervals on the exposed surface of the pile body to build a model training and verification database. The results show that the model can mine the dynamic characteristics of the pile foundation when it is forced to vibrate from the ideal data generated by the model, thereby completing the intelligent detection of pile foundation integrity.
- The calculation results of the models show that both architectures can effectively capture the main characteristic patterns in the signal, and the overall judgment results are highly accurate. Among them, the CNN model performs well in capturing fine-grained features due to its local receptive field characteristics, and its performance on the validation set is significantly better than that of the LSTM model.
- The longitudinal comparison of different data preprocessing methods under the isomorphic model shows that the input mode of the time series stacking method has a certain degree of improvement on the CNN model and the LSTM model. This significant improvement verifies the advantage of the sensor layout method of multi-point vibration detection in the intelligent detection of pile foundation damage.
- Combined with the results of horizontal and vertical comparison and error analysis, it is recommended to use a computational model based on the 2Dstack-CNN architecture to perform intelligent detection of the integrity of high-cap serving pile foundations using multi-sensor data fusion.

## Figures and Tables

**Table 1:** The variation ranges for each parameter.

Parameter	Unit	Variation Range	
		Min	Max
Pile length (*L_p_*)	m	20	30
Pile diameter	m	0.4	0.6
Pile material density	kg/m^3^	2400	2600
Poisson’s ratio of soil	-	0.25	0.35
Soil material density	kg/m^3^	1700	1900
Length of pile defect segment (*L_d_*)	m	1	3
Defect segment location	m	1.5	*L_p_*-*L_d_*-1.5
Pulse width	s	0.0015	0.003

**Table 2:** The results of the three performance indicators of the different calculation models.

Model	Dataset	*R* ^2^	MAE/(m)	VAF/%
1Dsplice-CNN	Training set	0.9980	0.1941	99.80
	Validation set	0.9894	0.4168	98.94
2Dstack-CNN	Training set	0.9992	0.1251	99.92
	Validation set	0.9988	0.1465	99.89
3Dtimefreq-CNN	Training set	0.9976	0.2169	99.76
	Validation set	0.9896	0.3834	98.96
1Dsplice-LSTM	Training set	0.9967	0.1985	99.52
	Validation set	0.9852	0.2984	98.57
2Dstack-LSTM	Training set	0.9943	0.2007	99.43
	Validation set	0.9821	0.3136	98.21

## Data Availability

The raw data supporting the conclusions of this article will be made available by the authors on request.
